# Mitochondrial oxidative phosphorylation in autosomal dominant optic atrophy

**DOI:** 10.1186/1471-2091-9-22

**Published:** 2008-09-10

**Authors:** Vladimir I Mayorov, Angela J Lowrey, Valerie Biousse, Nancy J Newman, Susan D Cline, Michael D Brown

**Affiliations:** 1 Division of Basic Medical Sciences, Mercer University School of Medicine, 1550 College St., Macon, GA 31207, USA; 2 Department of Ophthalmology and Neurology, Emory University School of Medicine, Emory Eye Center, 1365-B Clifton Road NE, Atlanta, GA 30322, USA; 3 The Coca-Cola Company, Coca-Cola Plaza, Atlanta, GA 30301, USA

## Abstract

**Background:**

Autosomal dominant optic atrophy (ADOA), a form of progressive bilateral blindness due to loss of retinal ganglion cells and optic nerve deterioration, arises predominantly from mutations in the nuclear gene for the mitochondrial GTPase, OPA1. OPA1 localizes to mitochondrial cristae in the inner membrane where electron transport chain complexes are enriched. While OPA1 has been characterized for its role in mitochondrial cristae structure and organelle fusion, possible effects of OPA1 on mitochondrial function have not been determined.

**Results:**

Mitochondria from six ADOA patients bearing *OPA1 *mutations and ten ADOA patients with unidentified gene mutations were studied for respiratory capacity and electron transport complex function. Results suggest that the nuclear DNA mutations that give rise to ADOA in our patient population do not alter mitochondrial electron transport.

**Conclusion:**

We conclude that the pathophysiology of ADOA likely stems from the role of OPA1 in mitochondrial structure or fusion and not from OPA1 support of oxidative phosphorylation.

## Background

Autosomal dominant optic atrophy (ADOA) is a progressive form of bilateral blindness that shares the end-stage clinical characteristics of retinal gangln cell (RGC) death and optic nerve atrophy with the mitochondrial disease, Leber's hereditary optic neuropathy (LHON) [1]. ADOA, also referred to as Kjer's disease (OMIM 165500), has an earlier onset than LHON, with patients presenting mainly during childhood or adolescence. Unlike LHON, which results from mutations in mitochondrial DNA (mtDNA)-encoded complex I subunits, ADOA disease results primarily from mutations in two nuclear genes, *OPA1 *at 3q28 and *OPA4 *at 18q12, and displays incomplete penetrance and variable expressivity in families [2-7]. *OPA1 *mutations are responsible for the majority of reported ADOA cases and nearly half of the reported disease mutations give rise to a truncated OPA1 protein [3]. *OPA1 *encodes a 960 amino acid mitochondrial dynamin-related GTPase that resides in the inner membrane cristae and plays an essential role in cristae structure and mitochondrial fusion [8-16]. However, the relationship between these roles for OPA1 and the relationship of either function to cellular energy production remain to be elucidated.

While *OPA1 *is critical for optic nerve function, the mechanism by which *OPA1 *mutations lead to blindness is unknown [1]. A few studies have found ADOA patient *OPA1 *defects to be associated with diminished ATP synthesis and aberrant mitochondrial architecture at the tissue and cellular level [17-19]. Since cellular structure and nuclear gene expression differs between neurons and most cell types used for analysis of ATP production in ADOA, *OPA1 *mutations may reduce cellular ATP synthesis by different molecular mechanisms in each cell type [17]. Without an analysis of isolated mitochondria from ADOA patients harboring *OPA1 *mutations, it remains unclear whether the reduced ATP production in cells arises from a biochemical defect in respiratory function or is secondary to a frank disruption of mitochondrial membrane morphology or mitochondrial biogenesis [17,20,21]. Due to its location in the cristae of the inner membrane, the OPA1 protein may support OXPHOS through interactions with electron transport chain complexes, by maintaining a membrane topology conducive to efficient electron transfer, or by facilitating the fusion of mitochondria into networks that are more responsive to cellular ATP demand. Haploinsufficiency for *OPA1 *has been suggested as the primary cause of heterozygote phenotypes based on the finding that a downregulation of *OPA1 *results in cristae disorganization and mitochondrial aggregation, however the level of OPA1 found in some ADOA patient cell lysates was near that of controls [19,20,22,23]. In our study, we examined mitochondria from patients harboring ADOA *OPA1 *mutations to determine whether the protein defects alter mitochondrial respiration.

In a previous publication, we reported seven novel pathological *OPA1 *mutations in a cohort of 30 patients diagnosed with ADOA [24]. Lymphoblast cell lines were established from blood samples taken from six of the seven patients bearing novel *OPA1 *disease variants (referred to as *OPA1*-positive) and from ten ADOA patients without *OPA1 *mutations (referred to as *OPA1*-negative) (see Table 1). From these patients, we have isolated and biochemically characterized lymphoblast mitochondria, as was done previously for LHON patients with primary complex I mutations, to determine whether the nuclear DNA mutations give rise to ADOA through defects in OXPHOS [25]. Our findings suggest that *OPA1 *does not directly contribute to mitochondrial OXPHOS, as neither respiratory capacity nor OXPHOS specific enzyme activity, were affected by the deleterious *OPA1 *variants. Likewise, mitochondrial function was not diminished in *OPA1*-negative ADOA lymphoblasts. Based on these findings, we conclude that *OPA1 *mutations that result in ADOA have indirect effects on ATP production that may correlate with *OPA1 *function in mitochondrial cristae structure or organelle fusion in RGCs.

**Table 1:** *OPA1 *mutations in *OPA1*-positive ADOA patients

**Patient**	**Gene Mutation**	**Exon**	**Protein Change**	**Protein Domain**
P2^*a*^	c.239A>G	2	Tyr80Cys	Mt targeting
	c.2883A>C^*b*^	28	Stop961Tyr	Coiled-coiled
P3	c.2522A>G	25	Tyr841Cys	C-terminal
P4	c.2780T>A	27	Leu927Stop^*c*^	Coiled-coil
P6	c.1654delT	17	Trp552fs^*c*^	Dynamin
P7	c.1929delC	20	Thr643fs^*c*^	Dynamin
P8	c.2708delTTAG	27	Val903fs^*c*^	Coiled-coil

^*a*^Compound heterozygote.

^*b*^Results in a 3 amino acid C-terminal extension.

^*c*^Mutations in *OPA1 *result in protein truncation at the amino acid shown.

## Methods

### Patient Cell Lines

All patients in this study were examined by two experienced neuro-ophthalmologists (NJN, VB) and had clinical symptoms, age of onset, and family histories typical for ADOA, as described previously [24]. Informed consent was obtained from each study participant using an institutional IRB approved consent form. *OPA1 *exons and intron/exon junctions were sequenced for all patients and a complete characterization of the ADOA-associated *OPA1 *variants have been described in detail in [24]. None harbored known LHON mtDNA mutations. Lymphoblast cell lines were established by EBV transformation of leukocytes isolated from whole blood by Ficoll-Hypaque gradient [25]. Lymphoblastoid cell lines were maintained in RPMI 1640 medium (Bio-Whitaker, Walkersville, MD) supplemented with 15% (v/v) heat-inactivated fetal bovine serum (Life Technologies, Inc., Grand Isle, NY).

### Mitochondrial Isolation, Polarography, and Enzymology

All procedures for mitochondrial isolation, polarographic respiration analysis and respiratory chain enzymology have been previously described [25-27]. Briefly, for respiration studies, electrons were entered at either complex I (using either malate + pyruvate or glutamate + malate as substrate) or complex II (using succinate as the substrate) using intact whole mitochondria. Two polarographic runs were performed for each substrate and each run included two additions of ADP (125 nmole for complex I substrates and 75 nmole for succinate) to stimulate state III and subsequent state IV respiration. Each run was concluded by the addition of the OXPHOS uncoupler, 2,4-dinitrophenol (DNP) to assess maximal respiration rates. All runs were performed with 250 – 500 μg of mitochondrial protein.

OXPHOS specific enzyme activities in submitochondrial particles were measured using a Varian Cary 300 Bio UV/Vis spectrophotometer [25-27]. Briefly, mitochondrial protein was prepared by sonication of isolated organelles. Complex I activity was monitored in triplicate samples as the reduction of 10 μM decylubiquinone at 272 nm by 30 μg of mitochondrial protein with 30 μM NADH. Using this method, 90–100% of the total complex I activity is sensitive to rotenone inhibition. Complex III and complex IV activities were determined at 550 nm in duplicate samples containing 7.5 μg mitochondrial protein and appropriate substrates. Complex III activity was assayed as the antimycin A-sensitive oxidation of reduced decylubiquinone by cytochrome c. Using this method, complex III activity is 75 – 100% antimycin A sensitive. Complex IV activity was determined by the cyanide-sensitive oxidation of cytochrome c. Citrate synthase was assayed at 412 nm in duplicate samples containing 15 μg of mitochondrial protein.

For all samples, mitochondrial protein concentration was determined using the modified Lowry assay and mitochondrial preparations were not used for biochemical analysis if the protein concentration was below 2 mg/ml. Further, all mitochondrial preparations exhibited a respiratory control ratio of ≥ 4, indicative of a high quality mitochondrial isolation as evidenced by well-coupled mitochondria upon polarographic analysis (*data not shown*) [25,28]. Statistical significance for all data (*p *< 0.05) was determined by the Mann-Whitney unpaired, two-tailed test using Instat Graphpad Software [25,29].

## Results

To assess mitochondrial function in ADOA patients, we determined mitochondrial respiratory function and respiratory chain enzyme specific activities in both *OPA1*-positive and *OPA1*-negative ADOA patients. For each patient cohort, both complex I-linked (malate + pyruvate and glutamate + malate substrates) and complex II-linked (succinate) maximal (state III) respiration rates were not significantly diminished relative to the control group (Table 2). For *OPA1*-positive patient mitochondria, only slight reductions in all complex I rates (ranging from 1–12%) were found when comparing the average rates from both complex I-linked substrates with the controls. These data suggest that OPA1 has no direct role in OXPHOS, however individual variation in OXPHOS exists within the *OPA1*-positive ADOA patient mitochondria. For example, mitochondria from patient P3 showed elevations in all respiration rates when entering electrons at complex I and complex II, and P6 mitochondria displayed a notable decrease in rates with all substrates. Although the data for individual patient mitochondria are insignificant due to sample size, results for P3 and P6 mutations suggest that further study may be warranted. For *OPA1*-negative patients, respiratory rate reductions were observed with complex I-linked substrates (ranging from 11–18%) and succinate (ranging from 15–20%), however these differences from the controls were not statistically significant. RCR values from polarography analysis revealed that all patient and control mitochondria were well-coupled, and ADP/O ratios showed no statistical difference between patients and controls for both complex I and complex II-linked substrates (*data not shown*). Overall, we could not detect respiration defects in *OPA1*-positive or -negative ADOA patient mitochondria.

**Table 2:** Polarographic analysis of ADOA patient and control mitochondria

	**Mean respiration rates and ADP/O with substrates indicated**^***a***^ ***nmol O/min/mg mitochondrial protein***								
	
	**Malate + Pyruvate**			**Glutamate + Malate**			**Succinate**		
			
	**III**	**IV**	**UC**	**III**	**IV**	**UC**	**III**	**IV**	**UC**
^*b*^OPA1+ (n = 6)									
P2	211	43	258	235	52	270	313	65	349
P3	349	58	442	398	50	474	537	97	653
P4	290	41	397	310	36	392	393	76	468
P6	173	36	280	170	31	276	276	54	372
P7	281	48	362	310	41	348	356	73	403
P8	250	44	322	280	35	338	310	57	391
Mean ± SD	259 ± 62	45 ± 8	344 ± 70	284 ± 77	41 ± 9	350 ± 76	364 ± 94	70 ± 16	439 ± 112
^*b*^OPA1- (n = 10)									
Mean ± SD	230 ± 64	38 ± 13	309 ± 99	270 ± 88	39 ± 18	312 ± 145	301 ± 83	58 ± 22	356 ± 124
Control (n = 10)									
Mean ± SD	300 ± 37	49 ± 12	370 ± 73	312 ± 38	45 ± 12	328 ± 98	376 ± 66	66 ± 18	421 ± 63

^*a*^Abbreviations used for respiratory rate data are III, state III rate; IV, state IV rate; and UC, uncoupled rate.

^*b*^Abbreviations used for patient genetics are OPA1+, *OPA1*-positive patients and OPA1-,*OPA1*-negative patients

Mitochondrial respiratory chain enzyme specific activities were assayed in submitochondrial particles for both patient groups (Table 3). Complexes I, III, IV, and the mitochondrial matrix enzyme citrate synthase (CS) were studied, with CS activity used for normalization of the respiratory chain enzyme activities. We found that complex I activity was reduced by 10.6% in *OPA1*-positive patients relative to controls, but this reduction was not statistically significant. The apparent reduction in complex I activity also was evident when complex I activities were normalized to CS activities, as *OPA1*-positive patients had a complex I/CS ratio of 0.11 while controls demonstrated a normalized ratio of 0.13. Thus, normalized complex I activities were still reduced in *OPA1*-positive patients, but by a modest 15%. Given the variability in the raw and CS normalized complex I activities, it remains possible that individual deleterious *OPA1 *mutations may impact OXPHOS and a careful analysis of a greater population of patients with of these mutations is needed. In *OPA1*-negative patients, complex I activity was essentially identical to controls (Table 3). For all patients, complex III and IV activities were very similar to the control activities.

**Table 3:** Specific enzyme analysis of ADOA patient and control mitochondria

	**Mean specific enzyme activities** ***nmol/min/mg mitochondrial protein***			
	
	**Complex I**	**Complex III**	**Complex IV**	**CS**^*a*^
^*a*^OPA1+ (n = 6)				
P2	82	1076	884	1410
P3	274	976	568	2074
P4	294	928	1005	1693
P6	86	1313	970	918
P7	52	1078	974	846
P8	122	845	828	1018
Mean ± SD	152 ± 105	1036 ± 162	872 ± 162	1327 ± 488
^*a*^OPA1- (n = 10)				
Mean ± SD	190 ± 144	945 ± 174	810 ± 204	1154 ± 650
Control (n = 10)				
Mean ± SD	170 ± 64	886 ± 179	893 ± 271	1304 ± 364

^*a*^Abbreviations used above are CS, citrate synthase, OPA1+, *OPA1*-positive patients, and OPA1-, *OPA1*-negative patients

For our *OPA1*-positive patients, two (P2 and P3) harbored pathogenic missense mutations and four (P4, P6-8) contained protein-truncating variants (Table 1). Since the protein terminating mutations may be considered potentially more severe than the missense mutations, we examined our data to see if this was the case from a functional perspective. Although our sample size is low, we found no significant differences between *OPA1*-positive patients with missense or frameshift/nonsense mutations. Further, patient P2 is a compound heterozygote, harboring both the c.239A>G exon 2 missense mutation and the c.2883A>C exon 28 variant that changes the *OPA1 *stop codon to a tyrosine and extends the OPA1 polypeptide 3 additional amino acids. As indicated in Tables 2 and 3 for *OPA1*-positive patient P2, both respiration and specific enzyme activities do not differ significantly when compared to the other *OPA1*-positive patients, suggesting that the combined presence of these two deleterious *OPA1 *variants does not cause greater functional impairment.

## Discussion

The analysis of six ADOA patient lymphoblastoid cell lines with mutations in the nuclear gene *OPA1 *indicates that *OPA1 *has no direct role in mitochondrial respiratory chain function. These data represent the first report on *in vitro *OXPHOS function using isolated mitochondria from patients with ADOA. No significant effects on respiratory capacity or specific electron transport chain enzyme activity were observed in mitochondria from *OPA1*-positive or *OPA1*-negative ADOA patients suggesting that ADOA-related gene defects alter mitochondrial function primarily by disruption of inner mitochondrial membrane topography. This is intriguing as the bilateral vision loss experienced by ADOA patients is clinically similar to that observed in LHON patients harboring primary complex I mtDNA variants that directly impair mitochondrial biochemistry [25,28]. Overall, it appears that RGC function is sensitive to different mechanisms of mitochondrial perturbation.

The study of *OPA1 *in the physiological context of ADOA is limited by the inability to culture patient RGCs. Similar to previous studies of LHON complex I mutations, our study of mitochondrial respiratory capacity of isolated ADOA patient organelles was an attempt to elucidate the pathological role of *OPA1 *at the biochemical level and enhance knowledge gained through several human cell models [17-19]. *OPA1 *is expressed as eight, tissue-specific transcripts resulting from alternative splicing in the gene region encompassing exons 4 through 5b [8,13]. *OPA1 *transcripts 1, 4 and 7 expressed in retina are also found in leukocytes, therefore the results obtained here with patient lymphoblast mitochondria should reflect any mitochondrial impairments due to the ADOA *OPA1 *mutations affecting protein isoforms in RGCs [8]. The protein products of *OPA1 *transcripts 1, 2, 4 and 7 support mitochondrial fusion activity to a greater extent than other OPA1 isoforms [30]. The OPA1 protein encoded by each transcript is cleaved by proteases within the mitochondrial matrix at sequences translated from exons 5 and 5b [13,30,31]. This activity removes the N-terminal, membrane-anchored region of the full-length protein generating one or more short, matrix isoforms. Both the long and short *OPA1 *gene products are required for mitochondrial fusion activity, and proteolysis is in part stimulated by loss of mitochondrial membrane potential [13,30].

The ADOA *OPA1 *mutations in our patient lymphoblasts alter key functional domains that are present in all OPA1 proteins and lie outside of the proteolytic sites of the OPA1 protein (Table 1). The mutations would affect both long and short isoforms and should disrupt OPA1-mediated networking of mitochondria that may be required for the high level of ATP production in RGCs [3,8-10,12,13,15,16,19,30]. Surprisingly, none of the *OPA1 *mutations evaluated here diminished OXPHOS to a significant extent, even though the c.2708delTTAG mutation has been associated with decreased ATP synthesis in fibroblasts and OPA1 appears to interact with AIF, a stability factor for electron transport chain complex I [19]. Although ADOA shares many clinical hallmarks of LHON, defects in *OPA1 *do not compromise mitochondrial respiratory capacity. These results suggest that *OPA1 *may not be required for maintaining the electron transport chain for OXPHOS and may function primarily at the level of mitochondrial morphology within RGCs. Indeed, *OPA1 *function in the control of morphology may require alterations in membrane potential or cellular signals not induced in the context of cell culture, thereby limiting our ability to observe *OPA1 *effects in isolated mitochondria.

A recent report by Schimpf *et al*. suggests that *OPA1 *mutations that would produce large C-terminal truncations in OPA1 protein may lead to message degradation by nonsense mediated mRNA decay (NMD) [32,33]. The net effect of NMD would be expression of protein only from the functional *OPA1 *allele, thereby creating a haploinsufficiency for *OPA1*. This mechanism may be the underlying cause of RGC death and disease in our *OPA1*-positive ADOA patients with two exceptions, patient P2 and patient P3. Patient P2 is a compound heterozygote harboring two mutant alleles, a mitochondrial targeting sequence mutation in exon 2 and a Stop961Tyr in exon 28, which extends the C-terminus by 3 amino acids. The exon 2 mutation should prevent mitochondrial localization of the gene product, resulting in a downregulation of functional protein expression and haploinsufficiency. The three amino acid extension of OPA1 is not expected to alter protein function, however a small effect on protein activity may be detrimental in the context of diminished levels mitochondrial OPA1. The *OPA1 *genotype of patient P3 is more intriguing, as the single base change results in an amino acid substitution, Tyr841Cys, that lies outside of known functional domains in the C-terminal region. The Tyr841Cys OPA1 protein should be expressed and targeted to the mitochondria where it conceivably may compete in molecular interactions with both the long and short isoforms of protein expressed from the normal allele. While no significance was found for the average mitochondrial respiration and specific activity data for the ADOA *OPA1*-positive mitochondria compared to controls, mitochondria from P3 lymphoblasts displayed notably higher respiration, slightly elevated complex I activity, and substantially lower complex IV activity. This suggests that the Tyr841 may mediate regulatory or stabilizing interactions with complexes I and IV within the mitochondrial membrane [18,19,34]. A more detailed analysis of the C-terminus of OPA1 may uncover critical interactions with the electron transport chain in the cristae of the mitochondria.

## Conclusion

From this study we conclude that *OPA1 *is not directly involved in maintaining electron transport efficiency for OXPHOS. The *OPA1 *mutations causing ADOA most likely result in a cellular haploinsufficiency for OPA1 protein in mitochondrial network formation and total cellular energy production. The results obtained for mitochondria bearing an OPA1 protein with a Tyr841Cys change in the C-terminus indicate that this domain of OPA1 could be responsible for stabilizing electron transport chain complexes and suggest that functional interactions for OPA1 may exist within the inner membrane. However, the apparent cause of ADOA pathology is the loss of OPA1 control of mitochondrial morphology in RGCs.

## Authors' contributions

VM isolated mitochondria from patient lymphoblasts, conducted all respiration and enzymology experiments, and performed data analysis. AL isolated DNA and prepared EBV-transformed lymphoblastoid lines from patient blood samples and maintained cell lines for mitochondrial isolation. VB and NN diagnosed patients and facilitated patient enrollment and collection of blood samples in accordance with the institutional IRB informed consent. SC performed statistical analysis and drafted and revised the manuscript. MB designed the study, supervised all data collection and analysis, and assisted in manuscript preparation. All authors read and approved submission of the final manuscript.
